# Dipotassium tetra­aqua­bis­(μ-citrato-κ^4^ *O*:*O*′,*O*′′,*O*′′′)nickelate(II) tetra­hydrate

**DOI:** 10.1107/S1600536813022630

**Published:** 2013-08-17

**Authors:** Hua-Gang Yao, Jia-Na Huang, Run-Kang Deng, Zhi-Bang Yao

**Affiliations:** a School of Chemistry and Chemical Engineering, Guangdong Pharmaceutical University, Guangdong 528458, People’s Republic of China

## Abstract

The title complex, K_2_[Ni_2_(C_6_H_5_O_7_)_2_(H_2_O)_4_]·4H_2_O, is a dinuclear centrosymmetric anionic octa­hedral complex, involv­ing citrates as tridentate and bridging ligands, and coordinating water mol­ecules. An extensive network of hydrogen bonds connects the complex anions through the two unique uncoordinating water mol­ecules. The K^+^ counter cation is surrounded by seven O atoms in the form of an irregular polyhedron and further stabilizes the crystal packing.

## Related literature

For applications of structures with metal-organic frameworks, see: Chui *et al.* (1999 ▶); Kahn & Martinez (1998 ▶); Kiang *et al.* (1999 ▶); Lin *et al.* (1999 ▶). For metal coordination polymers with a variety of topologies, see: Kondo *et al.* (2000 ▶); Shin *et al.* (2003 ▶); Wu *et al.* (2003 ▶); Yao *et al.* (2007 ▶). For the nickel–citrate complex K_2_[Ni(C_6_H_5_O_7_)(H_2_O)_2_]_2_·4H_2_O, which crystallized in the triclinic space group *P* 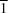, see: Baker *et al.* (1983 ▶).
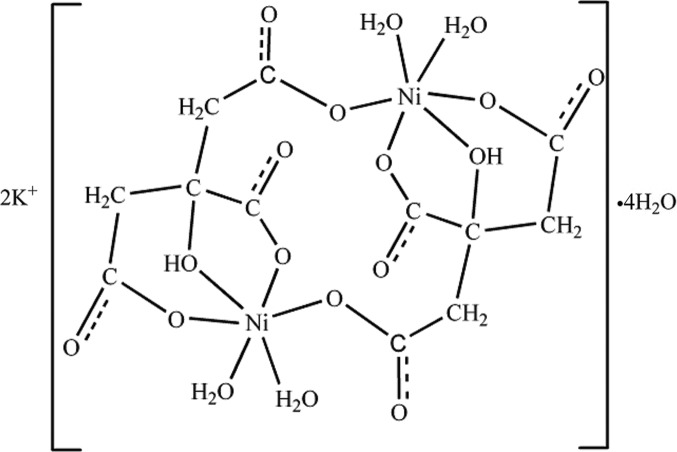


## Experimental

### 

#### Crystal data

K_2_[Ni_2_(C_6_H_5_O_7_)_2_(H_2_O)_4_]·4H_2_O
*M* *_r_* = 717.94
Monoclinic, 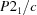

*a* = 10.616 (2) Å
*b* = 13.006 (3) Å
*c* = 9.0513 (18) Å
β = 93.09 (3)°
*V* = 1247.8 (4) Å^3^
*Z* = 2
Mo *K*α radiation
μ = 1.94 mm^−1^
*T* = 293 K
0.30 × 0.20 × 0.15 mm

#### Data collection

Bruker SMART APEXII CCD diffractometer
Absorption correction: multi-scan (*SADABS*; Sheldrick, 2001 ▶) *T*_min_ = 0.636, *T*_max_ = 0.741
9515 measured reflections
3128 independent reflections
2916 reflections with *I* > 2σ(*I*)
*R*_int_ = 0.017

#### Refinement

*R*[*F* ^2^ > 2σ(*F* ^2^)] = 0.023
*wR*(*F* ^2^) = 0.063
*S* = 1.02
3128 reflections
224 parameters
2 restraints
H-atom parameters constrained
Δρ_max_ = 0.43 e Å^−3^
Δρ_min_ = −0.55 e Å^−3^

### 

Data collection: *APEX2* (Bruker, 2004 ▶); cell refinement: *SAINT* (Bruker, 2004 ▶); data reduction: *SAINT*; program(s) used to solve structure: *SHELXS97* (Sheldrick, 2008 ▶); program(s) used to refine structure: *SHELXL97* (Sheldrick, 2008 ▶); molecular graphics: *SHELXTL* (Sheldrick, 2008 ▶); software used to prepare material for publication: *SHELXTL*.

## Supplementary Material

Crystal structure: contains datablock(s) global, I. DOI: 10.1107/S1600536813022630/kp2456sup1.cif

Structure factors: contains datablock(s) I. DOI: 10.1107/S1600536813022630/kp2456Isup2.hkl

Additional supplementary materials:  crystallographic information; 3D view; checkCIF report

## Figures and Tables

**Table 1:** Selected bond lengths (Å)

Ni1—O4	2.0322 (12)
Ni1—O2	2.0330 (11)
Ni1—O6	2.0345 (10)
Ni1—O2*W*	2.0677 (12)
Ni1—O1*W*	2.0709 (11)
Ni1—O3	2.0927 (10)

**Table 2:** Hydrogen-bond geometry (Å, °)

*D*—H⋯*A*	*D*—H	H⋯*A*	*D*⋯*A*	*D*—H⋯*A*
O1*W*—H1*WB*⋯O2^i^	0.79 (3)	1.96 (3)	2.7322 (16)	167 (2)
O2*W*—H2*WB*⋯O1^ii^	0.84 (3)	1.93 (3)	2.7638 (16)	170 (2)
O4*W*—H4*WB*⋯O5^iii^	0.85 (3)	1.91 (3)	2.7459 (17)	171 (2)
O2*W*—H2*WA*⋯O4*W* ^iv^	0.88 (3)	1.83 (3)	2.7064 (18)	174 (2)
O4*W*—H4*WA*⋯O5^v^	0.72 (3)	2.20 (2)	2.8714 (19)	155 (2)
O3—H1⋯O6^vi^	0.75 (2)	2.13 (2)	2.7152 (15)	135 (2)
O3*W*—H3*WA*⋯O2*W* ^vii^	0.85 (1)	2.25 (4)	2.912 (2)	135 (5)

Symmetry codes: (i) 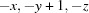; (ii) 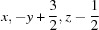; (iii) 
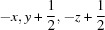
; (iv) 
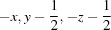
; (v) 
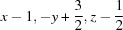
; (vi) 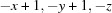; (vii) 
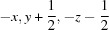
.

**Fig. 1.**
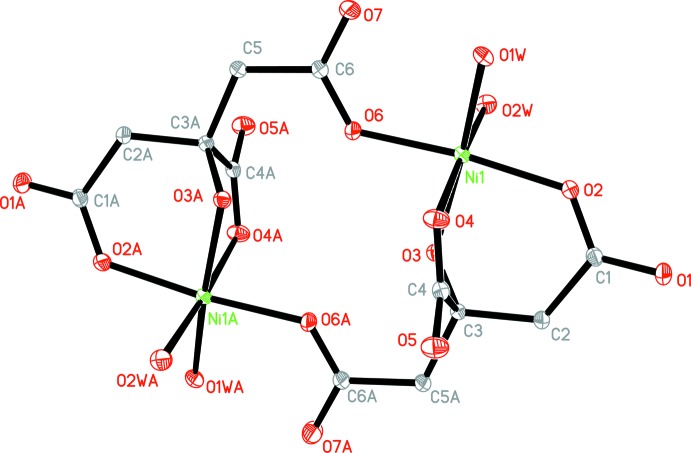
The structure of the dimeric complex anion, with the atom-labelling scheme. Displacement ellipsoids are drawn at the 30% probability level. H atoms have been omitted for clarity.

**Fig. 2.**
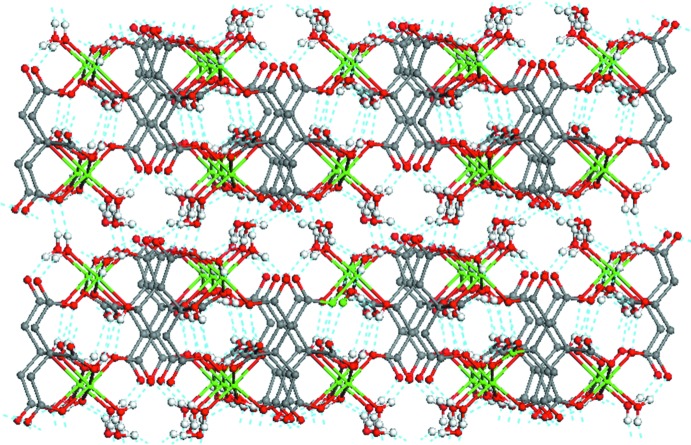
Dimeric complexes are consolidated into three-dimensional structures by hydrogen bonds. Symmetry code: -x, 1/2 + y, 1/2 - z

## supplementary crystallographic information

### 1. Comment

The construction of metal–organic frameworks (MOFs) is an area of intense research activity due to their intriguing structural diversity and potential applications as zeolitic, optoelectronic, magnetic and conducting materials (Chui *et al.*, 1999; Kiang *et al.*, 1999; Kahn & Martinez, 1998; Lin *et al.*, 1999). Depending on the conformation of carbon chains, the functional group of organic ligands and the type of metal ions, a variety of metal coordination polymers with different topological structures, such as one-dimensional chains (Shin *et al.*, 2003), two-dimensional grids (Kondo *et al.*, 2000), three-dimensional porous motifs (Yao *et al.*, 2007) and helical strands (Wu *et al.*, 2003) were observed. In this paper, we report the synthesis of a dimeric nickel(II) citrate complex by self-assembly under hydrothermal conditions.

In the crystal the centrosymmetric structural unit is a dinuclear Ni^II^ anion (Fig. 1) and the two potassium cations, and crystalline water molecules. The crystallographic unit is a half of the structural unit. The Ni^II^ ion adopts an octahedral coordination mode. One citrate ligand is bound with an hydroxyl and two carboxylate groups to the Ni^II^ ion, whereas one O atom (O6) from a carboxylate group of a symmetry-related citrate ligand occupies another apex, and two water molecules complete the octahedral environment. The Ni—O distances range from 2.0322 (12) Å to 2.0927 (10) Å (Table 1). Neighbouring dimeric complexes are consolidated into a three-dimensional structure by hydrogen bonds (Table 2, Fig. 2). The crystallographically independent potassium cation, K1, is seven-coordinated by O atoms, with an average contact distance of 2.852 Å.

The corresponding nickel–citrate complex with the triclinic space group P1, K_2_[Ni(C_6_H_5_O_7_)(H_2_O)_2_]_2_.4H_2_O, has been reported (Baker *et al.*, 1983). The complex exists as centrosymmetric dimers, which has identical structure with the title complex, but a difference is that the potassium ions and water molecules of crystallization occupy the spaces between the nickel–citrate dimers in the two cases, resulting in the different formation of the geometry of potassium ion and hydrogen bonds.

### 2. Experimental

Citric acid monohydrate (0.048 g), NiCl_2_.6H_2_O (0.042 g) and KOH (0.027 g) were dissolved in 6 ml mixed solvent of DMF–H_2_0 (2:1 *v*/*v*), which were placed in a small vial. The mixture was heated at 351 K for 3 d and then cooled to room temperature. Green block crystals of the product were collected by filtration and washed with ethanol several times (88% based on Ni). This synthetic route allowed us to obtain a pure phase. Elemental analysis, calculated (%) for title compound: C 20.06, H 3.62; found C 20.35, H 3.44.

### 3. Refinement

H atoms were positioned geometrically, with C—H = 0.93 Å, and allowed to ride during subsequent refinement, with *U*_iso_(H) = 1.2*U*_eq_(C).

### Crystal data

**Table:**

K_2_[Ni_2_(C_6_H_5_O_7_)_2_(H_2_O)_4_]·4H_2_O	*Z* = 2
*M**_r_* = 717.94	*F*(000) = 736
Monoclinic, *P*2_1_/*c*	*D*_x_ = 1.911 Mg m^−^^3^
Hall symbol: -P 2ybc	Mo *K*α radiation, λ = 0.71073 Å
*a* = 10.616 (2) Å	µ = 1.94 mm^−^^1^
*b* = 13.006 (3) Å	*T* = 293 K
*c* = 9.0513 (18) Å	Block, green
β = 93.09 (3)°	0.30 × 0.20 × 0.15 mm
*V* = 1247.8 (4) Å^3^	

### Data collection

**Table:**

Bruker SMART APEXII CCD diffractometer	3128 independent reflections
Radiation source: fine-focus sealed tube	2916 reflections with *I* > 2σ(*I*)
Graphite monochromator	*R*_int_ = 0.017
φ and ω scans	θ_max_ = 28.4°, θ_min_ = 1.9°
Absorption correction: multi-scan (*SADABS*; Sheldrick, 2001)	*h* = −14→12
*T*_min_ = 0.636, *T*_max_ = 0.741	*k* = −17→17
9515 measured reflections	*l* = −12→12

### Refinement

**Table:**

Refinement on *F*^2^	Primary atom site location: structure-invariant direct methods
Least-squares matrix: full	Secondary atom site location: difference Fourier map
*R*[*F*^2^ > 2σ(*F*^2^)] = 0.023	Hydrogen site location: inferred from neighbouring sites
*wR*(*F*^2^) = 0.063	H-atom parameters constrained
*S* = 1.02	*w* = 1/[σ^2^(*F*_o_^2^) + (0.0378*P*)^2^ + 0.4504*P*] where *P* = (*F*_o_^2^ + 2*F*_c_^2^)/3
3128 reflections	(Δ/σ)_max_ = 0.001
224 parameters	Δρ_max_ = 0.43 e Å^−^^3^
2 restraints	Δρ_min_ = −0.55 e Å^−^^3^

### Special details

**Table:**

Geometry. All e.s.d.'s (except the e.s.d. in the dihedral angle between two l.s. planes) are estimated using the full covariance matrix. The cell e.s.d.'s are taken into account individually in the estimation of e.s.d.'s in distances, angles and torsion angles; correlations between e.s.d.'s in cell parameters are only used when they are defined by crystal symmetry. An approximate (isotropic) treatment of cell e.s.d.'s is used for estimating e.s.d.'s involving l.s. planes.
Refinement. Refinement of *F*^2^ against ALL reflections. The weighted *R*-factor *wR* and goodness of fit *S* are based on *F*^2^, conventional *R*-factors *R* are based on *F*, with *F* set to zero for negative *F*^2^. The threshold expression of *F*^2^ > σ(*F*^2^) is used only for calculating *R*-factors(gt) *etc*. and is not relevant to the choice of reflections for refinement. *R*-factors based on *F*^2^ are statistically about twice as large as those based on *F*, and *R*-factors based on ALL data will be even larger.

### Fractional atomic coordinates and isotropic or equivalent isotropic displacement parameters (Å^2^)

**Table:**

	*x*	*y*	*z*	*U*_iso_*/*U*_eq_	
Ni1	0.243734 (14)	0.519202 (13)	−0.006123 (17)	0.01642 (7)	
K1	−0.13777 (3)	0.90086 (3)	0.14726 (4)	0.03020 (9)	
O1	0.07698 (9)	0.76820 (8)	0.19256 (13)	0.0290 (2)	
O2	0.12630 (9)	0.63014 (8)	0.06472 (12)	0.0256 (2)	
O3	0.38530 (9)	0.63022 (8)	−0.02002 (10)	0.01756 (18)	
O4	0.32413 (10)	0.51024 (8)	0.20239 (12)	0.0247 (2)	
O5	0.43183 (11)	0.60531 (8)	0.37004 (11)	0.0278 (2)	
O6	0.37540 (9)	0.41901 (8)	−0.07541 (12)	0.0240 (2)	
O7	0.25885 (11)	0.27918 (10)	−0.11481 (19)	0.0503 (4)	
O1W	0.11765 (10)	0.40268 (8)	0.03601 (12)	0.0235 (2)	
O2W	0.16893 (11)	0.54135 (9)	−0.21942 (12)	0.0238 (2)	
O3W	−0.00587 (17)	0.89783 (14)	−0.11374 (17)	0.0549 (4)	
O4W	−0.32069 (13)	0.97832 (11)	−0.06745 (14)	0.0336 (3)	
C1	0.15547 (12)	0.71310 (10)	0.13242 (14)	0.0187 (2)	
C2	0.29093 (12)	0.75171 (10)	0.13945 (16)	0.0202 (3)	
C3	0.39866 (11)	0.67514 (10)	0.12633 (13)	0.0160 (2)	
C4	0.38568 (12)	0.58926 (10)	0.24245 (14)	0.0181 (2)	
C5	0.47751 (12)	0.26558 (11)	−0.14594 (16)	0.0197 (3)	
C6	0.36067 (12)	0.32483 (11)	−0.11019 (15)	0.0212 (3)	
H1	0.444 (2)	0.6015 (17)	−0.036 (2)	0.040 (6)*	
H2A	0.2987 (17)	0.8014 (15)	0.067 (2)	0.030 (5)*	
H2B	0.3039 (17)	0.7872 (16)	0.227 (2)	0.031 (5)*	
H5A	0.4817 (18)	0.2059 (16)	−0.090 (2)	0.033 (5)*	
H5B	0.4680 (17)	0.2407 (15)	−0.249 (2)	0.029 (5)*	
H1WB	0.047 (2)	0.4026 (18)	0.003 (2)	0.048 (6)*	
H1WA	0.152 (2)	0.354 (2)	−0.006 (3)	0.065 (8)*	
H2WA	0.220 (2)	0.525 (2)	−0.290 (3)	0.057 (7)*	
H2WB	0.140 (2)	0.601 (2)	−0.235 (3)	0.055 (7)*	
H3WC	−0.003 (3)	0.8359 (11)	−0.141 (4)	0.096 (11)*	
H3WA	−0.073 (3)	0.906 (4)	−0.168 (5)	0.18 (2)*	
H4WA	−0.372 (2)	0.948 (2)	−0.100 (3)	0.041 (6)*	
H4WB	−0.351 (2)	1.0134 (19)	0.001 (3)	0.053 (7)*	

### Atomic displacement parameters (Å^2^)

**Table:**

	*U*^11^	*U*^22^	*U*^33^	*U*^12^	*U*^13^	*U*^23^
Ni1	0.01296 (10)	0.01699 (11)	0.01930 (10)	−0.00081 (6)	0.00068 (6)	−0.00159 (6)
K1	0.02800 (17)	0.03044 (18)	0.03236 (17)	0.00154 (13)	0.00338 (13)	−0.00315 (13)
O4	0.0277 (5)	0.0229 (5)	0.0230 (5)	−0.0075 (4)	−0.0032 (4)	0.0043 (4)
O3	0.0162 (4)	0.0189 (4)	0.0176 (4)	0.0000 (4)	0.0014 (3)	−0.0016 (3)
O6	0.0158 (4)	0.0174 (5)	0.0390 (6)	0.0003 (4)	0.0041 (4)	−0.0049 (4)
O2	0.0151 (4)	0.0257 (5)	0.0358 (5)	0.0002 (4)	0.0014 (4)	−0.0107 (4)
O5	0.0335 (6)	0.0305 (6)	0.0188 (5)	−0.0038 (4)	−0.0047 (4)	−0.0002 (4)
O1	0.0182 (5)	0.0257 (5)	0.0438 (6)	0.0019 (4)	0.0077 (4)	−0.0090 (5)
C5	0.0148 (6)	0.0170 (6)	0.0273 (7)	−0.0008 (5)	0.0012 (5)	−0.0021 (5)
C6	0.0155 (6)	0.0201 (6)	0.0281 (6)	−0.0004 (5)	0.0018 (5)	−0.0029 (5)
C2	0.0152 (6)	0.0169 (6)	0.0286 (7)	0.0014 (5)	0.0011 (5)	−0.0030 (5)
O7	0.0178 (5)	0.0321 (7)	0.1024 (12)	−0.0080 (5)	0.0151 (6)	−0.0266 (7)
C1	0.0149 (6)	0.0201 (6)	0.0209 (6)	0.0015 (5)	0.0006 (4)	0.0004 (5)
C3	0.0136 (5)	0.0167 (6)	0.0175 (5)	0.0004 (4)	0.0004 (4)	−0.0021 (4)
C4	0.0147 (6)	0.0196 (6)	0.0201 (6)	0.0019 (5)	0.0009 (4)	0.0005 (5)
O1W	0.0150 (5)	0.0245 (5)	0.0308 (5)	−0.0034 (4)	0.0016 (4)	−0.0013 (4)
O2W	0.0265 (5)	0.0223 (5)	0.0224 (5)	−0.0007 (4)	−0.0003 (4)	0.0012 (4)
O3W	0.0621 (10)	0.0611 (10)	0.0421 (8)	0.0235 (8)	0.0082 (7)	−0.0023 (7)
O4W	0.0313 (6)	0.0412 (7)	0.0287 (6)	−0.0052 (5)	0.0048 (5)	−0.0084 (5)

### Geometric parameters (Å, º)

**Table:**

Ni1—O4	2.0322 (12)	O1—C1	1.2462 (16)
Ni1—O2	2.0330 (11)	C5—C6	1.5100 (18)
Ni1—O6	2.0345 (10)	C5—C3^v^	1.5259 (18)
Ni1—O2W	2.0677 (12)	C5—H5B	0.984 (18)
Ni1—O1W	2.0709 (11)	C5—H5A	0.93 (2)
Ni1—O3	2.0927 (10)	C6—O7	1.2320 (17)
K1—O7^i^	2.6796 (13)	C2—C1	1.5213 (18)
K1—O3W	2.8108 (17)	C2—C3	1.5259 (17)
K1—O4^ii^	2.8425 (12)	C2—H2B	0.925 (19)
K1—O4W	2.8557 (16)	C2—H2A	0.926 (19)
K1—O1W^ii^	2.8633 (13)	O7—K1^i^	2.6796 (13)
K1—O1	2.8708 (12)	C3—C5^v^	1.5259 (18)
K1—O3W^iii^	3.053 (2)	C3—C4	1.5449 (18)
O4—C4	1.2606 (17)	O1W—K1^iv^	2.8633 (13)
O4—K1^iv^	2.8425 (12)	O1W—H1WB	0.79 (3)
O3—C3	1.4477 (15)	O1W—H1WA	0.83 (3)
O3—H1	0.75 (2)	O4W—H4WB	0.85 (3)
O6—C6	1.2723 (17)	O4W—H4WA	0.72 (3)
O2W—H2WB	0.84 (3)	O3W—K1^iii^	3.053 (2)
O2W—H2WA	0.88 (3)	O3W—H3WC	0.842 (10)
O2—C1	1.2707 (17)	O3W—H3WA	0.845 (10)
O5—C4	1.2475 (17)		
			
O4—Ni1—O2	88.95 (5)	C1—O2—Ni1	128.10 (9)
O4—Ni1—O6	89.34 (5)	C1—O1—K1	145.86 (10)
O2—Ni1—O6	174.20 (4)	C6—C5—C3^v^	115.44 (11)
O4—Ni1—O2W	174.86 (4)	C6—C5—H5B	109.1 (11)
O2—Ni1—O2W	89.11 (5)	C3^v^—C5—H5B	108.7 (11)
O6—Ni1—O2W	92.12 (5)	C6—C5—H5A	109.1 (12)
O4—Ni1—O1W	91.73 (5)	C3^v^—C5—H5A	110.1 (12)
O2—Ni1—O1W	92.74 (5)	H5B—C5—H5A	103.7 (16)
O6—Ni1—O1W	92.85 (5)	O7—C6—O6	124.63 (13)
O2W—Ni1—O1W	93.12 (5)	O7—C6—C5	118.43 (13)
O4—Ni1—O3	80.12 (4)	O6—C6—C5	116.93 (12)
O2—Ni1—O3	89.07 (5)	C1—C2—C3	119.47 (11)
O6—Ni1—O3	85.17 (4)	C1—C2—H2B	107.3 (12)
O2W—Ni1—O3	95.08 (4)	C3—C2—H2B	108.4 (12)
O1W—Ni1—O3	171.62 (4)	C1—C2—H2A	108.7 (11)
O7^i^—K1—O3W	98.82 (5)	C3—C2—H2A	107.8 (11)
O7^i^—K1—O4^ii^	98.49 (4)	H2B—C2—H2A	104.2 (16)
O3W—K1—O4^ii^	143.18 (4)	C6—O7—K1^i^	147.10 (10)
O7^i^—K1—O4W	85.94 (5)	O1—C1—O2	123.20 (12)
O3W—K1—O4W	77.54 (5)	O1—C1—C2	116.40 (12)
O4^ii^—K1—O4W	71.58 (4)	O2—C1—C2	120.38 (11)
O7^i^—K1—O1W^ii^	97.26 (5)	O3—C3—C2	107.32 (10)
O3W—K1—O1W^ii^	145.89 (5)	O3—C3—C5^v^	110.58 (10)
O4^ii^—K1—O1W^ii^	62.15 (4)	C2—C3—C5^v^	107.80 (11)
O4W—K1—O1W^ii^	133.61 (4)	O3—C3—C4	108.84 (10)
O7^i^—K1—O1	82.13 (4)	C2—C3—C4	108.91 (10)
O3W—K1—O1	71.57 (5)	C5^v^—C3—C4	113.21 (11)
O4^ii^—K1—O1	143.15 (3)	O5—C4—O4	125.05 (13)
O4W—K1—O1	144.57 (4)	O5—C4—C3	117.64 (12)
O1W^ii^—K1—O1	81.15 (4)	O4—C4—C3	117.23 (11)
O7^i^—K1—O3W^iii^	167.85 (5)	Ni1—O1W—K1^iv^	100.16 (5)
O3W—K1—O3W^iii^	69.78 (6)	Ni1—O1W—H1WB	122.5 (17)
O4^ii^—K1—O3W^iii^	89.00 (4)	K1^iv^—O1W—H1WB	113.6 (16)
O4W—K1—O3W^iii^	87.41 (5)	Ni1—O1W—H1WA	99.7 (18)
O1W^ii^—K1—O3W^iii^	94.69 (4)	K1^iv^—O1W—H1WA	116.3 (18)
O1—K1—O3W^iii^	97.62 (4)	H1WB—O1W—H1WA	104 (2)
C4—O4—Ni1	113.92 (9)	K1—O4W—H4WB	87.9 (17)
C4—O4—K1^iv^	129.32 (9)	K1—O4W—H4WA	123.4 (19)
Ni1—O4—K1^iv^	101.83 (4)	H4WB—O4W—H4WA	107 (2)
C3—O3—Ni1	104.99 (7)	K1—O3W—K1^iii^	110.22 (6)
C3—O3—H1	109.4 (17)	K1—O3W—H3WC	107 (2)
Ni1—O3—H1	106.2 (17)	K1^iii^—O3W—H3WC	140 (2)
C6—O6—Ni1	128.00 (9)	K1—O3W—H3WA	92 (3)
Ni1—O2W—H2WB	114.0 (16)	K1^iii^—O3W—H3WA	104 (4)
Ni1—O2W—H2WA	115.0 (16)	H3WC—O3W—H3WA	90 (4)
H2WB—O2W—H2WA	109 (2)		

Symmetry codes: (i) −*x*, −*y*+1, −*z*; (ii) −*x*, *y*+1/2, −*z*+1/2; (iii) −*x*, −*y*+2, −*z*; (iv) −*x*, *y*−1/2, −*z*+1/2; (v) −*x*+1, −*y*+1, −*z*.

### Hydrogen-bond geometry (Å, º)

**Table:**

*D*—H···*A*	*D*—H	H···*A*	*D*···*A*	*D*—H···*A*
O1*W*—H1*WB*···O2^i^	0.79 (3)	1.96 (3)	2.7322 (16)	167 (2)
O2*W*—H2*WB*···O1^vi^	0.84 (3)	1.93 (3)	2.7638 (16)	170 (2)
O4*W*—H4*WB*···O5^ii^	0.85 (3)	1.91 (3)	2.7459 (17)	171 (2)
O2*W*—H2*WA*···O4*W*^vii^	0.88 (3)	1.83 (3)	2.7064 (18)	174 (2)
O4*W*—H4*WA*···O5^viii^	0.72 (3)	2.20 (2)	2.8714 (19)	155 (2)
O3—H1···O6^v^	0.75 (2)	2.13 (2)	2.7152 (15)	135 (2)
O3*W*—H3*WA*···O2*W*^ix^	0.85 (1)	2.25 (4)	2.912 (2)	135 (5)

Symmetry codes: (i) −*x*, −*y*+1, −*z*; (ii) −*x*, *y*+1/2, −*z*+1/2; (v) −*x*+1, −*y*+1, −*z*; (vi) *x*, −*y*+3/2, *z*−1/2; (vii) −*x*, *y*−1/2, −*z*−1/2; (viii) *x*−1, −*y*+3/2, *z*−1/2; (ix) −*x*, *y*+1/2, −*z*−1/2.

## References

[bb1] Baker, E. N., Baker, H. N., Anderson, B. F. & Reevs, R. D. (1983). *Inorg. Chim. Acta*, **78**, 281–285.

[bb2] Bruker (2004). *APEX2* and *SAINT* Bruker AXS Inc., Madison, Wisconsin, USA.

[bb3] Chui, S. S. Y., Lo, S. M. F., Charmant, J. P. H., Orpen, A. G. & Williams, I. D. (1999). *Science*, **283**, 1148–1150. 10.1126/science.283.5405.1148 10024237

[bb4] Kahn, O. & Martinez, C. J. (1998). *Science*, **279**, 44–46.

[bb5] Kiang, Y.-H., Gardner, G. B., Lee, S., Xu, Z. & Lobkovsky, E. B. (1999). *J. Am. Chem. Soc.* **121**, 8204–8206. 10.1021/ja0115518 11772069

[bb6] Kondo, M., Shimamura, M., Noro, S. I., Minakoshi, S., Asami, A., Seki, K. & Kitagawa, S. (2000). *Chem. Mater.* **12**, 1288–1295.

[bb7] Lin, W., Wang, Z. & Ma, L.-J. (1999). *J. Am. Chem. Soc.* **121**, 11249–11251.

[bb8] Sheldrick, G. M. (2001). *SADABS* University of Gottingen, Germany.

[bb9] Sheldrick, G. M. (2008). *Acta Cryst.* A**64**, 112–122. 10.1107/S0108767307043930 18156677

[bb10] Shin, D.-M., Lee, I.-S., Lee, Y.-A. & Chung, Y.-K. (2003). *Inorg. Chem.* **42**, 2977–2981. 10.1021/ic0260163 12716190

[bb11] Wu, C.-D., Lu, C.-Z., Lin, X., Wu, D.-M., Lu, S.-F., Zhang, H.-H. & Huang, J.-S. (2003). *Chem. Commun.* pp. 1254–1255.

[bb12] Yao, H.-G., Ji, M., Ji, S.-H., Jiang, Y.-S., Li, L. & An, Y.-L. (2007). *Inorg. Chem. Commun.* **10**, 440–442.

